# Spatially Resolved Immunometabolism to Understand Infectious Disease Progression

**DOI:** 10.3389/fmicb.2021.709728

**Published:** 2021-08-19

**Authors:** Roel Tans, Shoumit Dey, Nidhi Sharma Dey, Grant Calder, Peter O’Toole, Paul M. Kaye, Ron M. A. Heeren

**Affiliations:** ^1^Division of Imaging Mass Spectrometry, Maastricht Multimodal Molecular Imaging (M4I) Institute, Maastricht University, Maastricht, Netherlands; ^2^Hull York Medical School, York Biomedical Research Institute, University of York, York, United Kingdom; ^3^Department of Biology, University of York, York, United Kingdom

**Keywords:** immunometabolism, infectious disease, inflammation, granulomas, mass spectrometry imaging, spatial transcriptomics, multi-*omics*, leishmaniasis

## Abstract

Infectious diseases, including those of viral, bacterial, fungal, and parasitic origin are often characterized by focal inflammation occurring in one or more distinct tissues. Tissue-specific outcomes of infection are also evident in many infectious diseases, suggesting that the local microenvironment may instruct complex and diverse innate and adaptive cellular responses resulting in locally distinct molecular signatures. In turn, these molecular signatures may both drive and be responsive to local metabolic changes in immune as well as non-immune cells, ultimately shaping the outcome of infection. Given the spatial complexity of immune and inflammatory responses during infection, it is evident that understanding the spatial organization of transcripts, proteins, lipids, and metabolites is pivotal to delineating the underlying regulation of local immunity. Molecular imaging techniques like mass spectrometry imaging and spatially resolved, highly multiplexed immunohistochemistry and transcriptomics can define detailed metabolic signatures at the microenvironmental level. Moreover, a successful complementation of these two imaging techniques would allow multi-*omics* analyses of inflammatory microenvironments to facilitate understanding of disease pathogenesis and identify novel targets for therapeutic intervention. Here, we describe strategies for downstream data analysis of spatially resolved multi-*omics* data and, using leishmaniasis as an exemplar, describe how such analysis can be applied in a disease-specific context.

## Introduction

Metabolism reflects an intrinsically complex interplay between multiple molecular and cellular networks. These dynamic networks underlie essential biological functions such as inflammatory responses, tissue degeneration, and regeneration. Notably, metabolism has an intimate relationship with immune cell activation and underpins many of the well-defined activation and differentiation states associated with cells of the innate and acquired immune response. For example, studies of macrophage polarization under the influence of LPS and interferon-γ (IFN-γ) or IL-4 [generating M1 and M2 phenotypes, respectively (Murray et al., 2014)] have revealed their strong association with glycolysis (van den Bossche et al., 2012; Tannahill et al., 2013; Palsson-McDermott et al., 2015; Millet et al., 2016; Shirai et al., 2016; Xie et al., 2016) and oxidative phosphorylation (Vats et al., 2006; Van den Bossche et al., 2016), respectively. Integrating *in vitro* metabolic and transcriptional data on macrophage polarization has revealed how metabolism is affected between polar macrophage activation (Jha et al., 2015). Similarly, differentiation of CD4^+^ T cell effector function is associated with the regulation of aerobic glycolysis (Chang et al., 2013). Whilst *in vitro* studies provide clear proof of concept and underscore a link between metabolism and immune function, they may fail to sufficiently account for the more complex and dynamic interactions that occur *in vivo* in the context of acute and chronic inflammatory microenvironments.

Inflammation reflects the coordinated aggregation of immune cells in response to tissue injury, infection, or other insults that disrupt tissue homeostasis. Inflammatory foci typically contain a wide variety of myeloid and lymphoid cell subsets, with composition varying in a dynamic manner from initiation to resolution. The biology of inflammation has for many years been studied using whole tissue approaches to evaluate gene and protein expression [e.g., whole tissue transcriptomics] or through the use of techniques that require tissue dissociation [e.g., flow cytometry (Leung et al., 1985) or scRNA-seq (Satija and Shalek, 2014)]. Additionally, the advent of dual RNA sequencing of both the host and pathogen has provided further insights into metabolic divergence of different macrophage lineages and pathogen fitness amongst these lineages during infection-associated inflammation (Pisu et al., 2020). Nevertheless, it is becoming increasingly appreciated that a full understanding of the processes governing inflammation requires a spatial context and this is likely to be equally true for studies aiming to delineate and understand the underlying metabolic signatures of immune cells at inflammatory sites.

Analysis of the molecular composition of a tissue is pivotal in understanding the basis of a broad spectrum of disease mechanisms and to determine appropriate diagnostics (Norris and Caprioli, 2013; Aichler and Walch, 2015). In clinical research and diagnostics, medical imaging of tissues is an indispensable technique. Classical hematoxylin and eosin (H&E) staining of tissues reveals tissue histology whereas medical images generated by magnetic resonance imaging (MRI) or computed tomography (CT) reveal tissue morphology and anatomy and enable monitoring of dynamic organ functions in health and disease using high-resolution tomographic images (Porta Siegel et al., 2018).

Technological advances have enabled multi-modal and multi-omics analyses to study the intrinsic interplay of genes, proteins, lipids, and metabolites in single cells which was recognized by Nature as the method of the year 2019 (Single-cell multimodal omics, 2020). Complementing these multi-modal and multi-omics methodologies to investigate immunometabolism is promising, yet they come with a variety of challenges. Two main challenges, which is reviewed elsewhere (Artyomov and van den Bossche, 2020; Lercher et al., 2020; Mazumdar et al., 2020), are to preserve the spatial context of biomolecules and the downstream analysis of the different *omes*.

Here, we provide a perspective on how multi-modal label-free molecular imaging techniques such as mass spectrometry imaging (MSI) combined with spatially resolved highly multiplexed protein and transcriptome mapping can be used in concert to understand the progression of infectious diseases and delineate inflammatory microenvironments. We discuss strategies for downstream data integration and analysis and describe a case study of research on granulomatous inflammation associated with experimental leishmaniasis as a possible exemplar of how research into inflammatory diseases of infectious (as well as non-infectious) origin can benefit from this approach.

### Mass Spectrometry Imaging

Mass spectrometry (MS) is a technique that measures the mass of atoms and molecules (Chughtai and Heeren, 2010). The specificity of MS allows structural analysis of small and large biomolecules, like metabolites, lipids, or large protein complexes, resulting in unambiguous molecular compound identifications (McDonnell and Heeren, 2007). In addition, rapid advancements of MS-related methodologies have increased its use in a wide variety of (bio)medical sciences, like infectious diseases (Seng et al., 2010; Zheng et al., 2010), oncology (Ifa and Eberlin, 2016; Takats et al., 2017; Mas et al., 2020), pharmacology (Jove et al., 2019), personalized medicine (Chen et al., 2012; Geyer et al., 2016), and more.

Initially described by Caprioli et al. (1997), MSI translates the spatial molecular context of tissues into improved understanding of biological processes underlying health and disease (Chughtai and Heeren, 2011; Porta Siegel et al., 2018). MSI uses an ionization technique that generates charged molecules (ions) from which the mass-to-charge ratios of ions can be determined (Chughtai and Heeren, 2010). Several ionization techniques exist like matrix-assisted laser desorption/ionization (MALDI), desorption electrospray ionization (DESI), and secondary ion mass spectrometry (SIMS) as well as different types of mass analyzers. It is important to recognize that the combination of ionization technique and type of mass analyzer depicts the sensitivity, specificity, and overall spatial resolution of an MSI workflow. In addition, the biomolecule class (i.e., protein, lipid, or metabolite) spatially analyzed in MSI is dependent on sample pre-treatment.

Mass spectrometry imaging defines the molecular composition at the tissue level and even at cellular level, resulting in improved understanding of the tissue microenvironment and can concomitantly identify specific molecular profiles of disease (Ščupáková et al., 2020). Moreover, it facilitates the translation of molecular images to the pathology of tissues (Norris and Caprioli, 2013; Schwamborn et al., 2017). This translation to pathology is enabled by the ability to align histological images, e.g., following H&E staining or immunohistochemistry (IHC), with MSI-generated images. As described earlier (Deutskens et al., 2011), post MALDI-MSI allows classical histological staining and microscopic evaluation of the same tissue section. Such combination of histology and MSI enables the study of molecular patterns associated with specific cell types or tissue structures (Porta Siegel et al., 2018).

A recent development combined MSI with laser microdissection and sensitive liquid chromatography-mass spectrometry (LC-MS) based proteomics. This application uses MSI-guided evaluation of (micro) regions of interest (ROIs) followed by targeted extraction of cellular material and conventional LC-MS/MS proteomics. As a result, MSI-guided spatial *omics* allows for a more in-depth molecular characterization of a tissue microenvironment (Dewez et al., 2020).

### Spatially Resolved, Highly Multiplexed Immunohistochemistry, and Transcriptomics

Spatial transcriptomic and multiplexed IHC can provide a transcriptional and functional snapshot of an inflammatory microenvironment. Profiling such *in situ* microenvironments can be achieved by a variety of techniques. McCaffrey et al. (2020) used multiplexed ion beam imaging by time of flight (MIBI-TOF) to spatially map and comprehensively delineate lung granulomas in patients with tuberculosis into cellular niches. Using individually metal isotope tagged antibodies and imaging based on SIMS, they found that the innermost core of granulomas is rich in myeloid suppressor cells exhibiting high expression of immunomodulatory immune checkpoint molecules IDO-1 and PD-L1 (McCaffrey et al., 2020). The 10× Genomics Visium platform that allows transcriptomic mapping of whole tissue sections using spatially bar-coded capture spots was used in combination with scRNA-seq to delineate granuloma architecture and show that non-immune cells can also produce antimicrobial factors that limit infection in leprosy (Ma et al., 2021). In another study (Carow et al., 2019) *in situ* mRNA sequencing was used to study murine lung granuloma progression over time. Other platforms like Hyperion that marry CyTOF technology and imaging mass cytometry (IMC) have recently been used to spatially map immune signatures and study lung pathology in COVID-19 (Zhang et al., 2020; Butler et al., 2021; Chevrier et al., 2021). Another key technology CODEX uses iterative cycles of imaging and removal of reporters that bind to complementary barcodes on antibodies used to stain tissue sections. It has been exploited extensively in cancer research (Parra et al., 2019; Schürch et al., 2020) and may be used to probe immune microenvironments.

While the above-named technologies commonly scan an entire tissue section, the Nanostring GeoMx Digital Spatial Profiler (DSP) platform (Merritt et al., 2020) allows more precise selection of user-defined regions of interest by using a unique method of patterned UV light (Merritt et al., 2020). Further, it allows segmentation analysis based on morphology markers for profiling cell types of particular interest. A typical workflow involves staining tissue sections with oligonucleotide tagged antibodies or RNA probes, histological, or fluorescently labeled image guided ROI selection, UV cleavage of tags specifically from the whole ROI or from a segment within an ROI, collection, and finally, barcode counting in the nCounter system (or next gen sequencing). This ability to select biologically relevant regions becomes particularly relevant when DSP is used to assess specific microenvironments, as applied recently in studies of cutaneous leishmaniasis (Dey et al., 2021) and COVID-19 pathology (Margaroli et al., 2021; Rendeiro et al., 2021).

## Downstream Analysis of Multi-Omics Data

**FIGURE 1:**
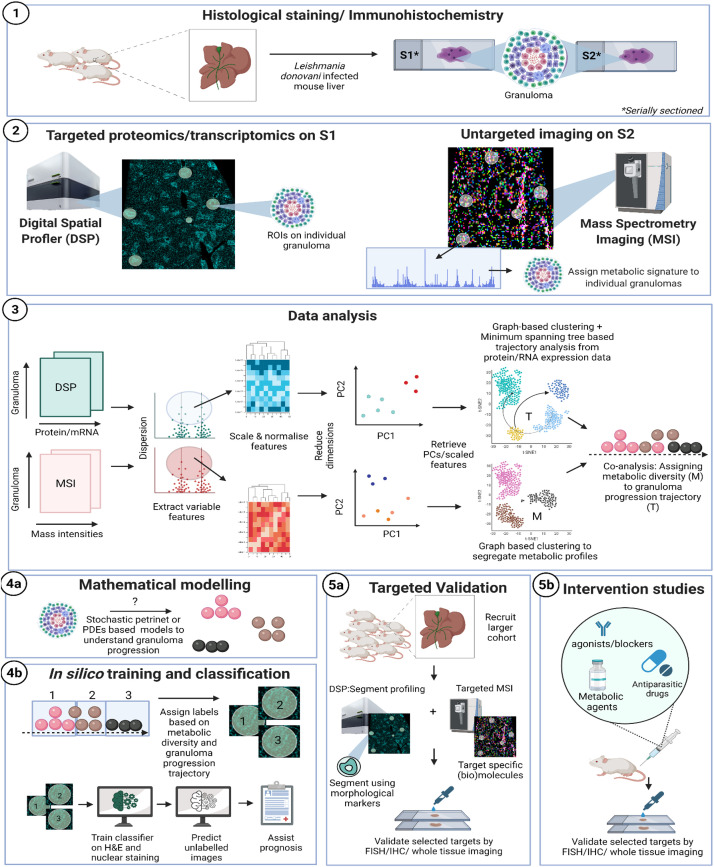
Proposed workflow: **(1)** classical hematoxylin and eosin (H&E) or nuclear staining of a mouse liver, infected with *Leishmania donovani* can be used to select granulomatous regions of interest (ROI). **(2)** Thin serial sections (5 μm) processed in parallel through GeoMx Digital Spatial Profiler (DSP; left) and mass spectroscopy imaging (MSI; right). For DSP: targeted morphology-based selection of ROIs can be probed using antibody-based protein profiling (e.g., mouse Immune Panel, NanoString). In parallel, MSI is used to analyze the whole tissue section at a predefined spatial resolution, typically 10–50 μm pixel size. MSI imaging is used to generate the molecular masses of biomolecules (e.g., of lipids, metabolites, or proteins), depending on the sample’s pretreatment, from each pixel. MSI images can be either co-registered with the corresponding DSP-imaged area based on H&E/nuclear staining. **(3)** Data obtained from DSP and untargeted MSI imaging are processed in parallel to extract most variable features, which are then scaled, normalized, and finally visualized in reduced dimensions to assess microenvironment (ROI) heterogeneity through PCA. Principal components that explain most of the variance in the data are used to cluster granuloma subtypes based on proteomic/transcriptomic data (DSP) or their metabolic profile (MSI). Progression trajectory inferred from transcriptomic data is overlaid on metabolic signature to identify aberrant lipids as an example. **(4a)** A mathematical re-construction of granuloma progression and its association to cell types, chemokine/cytokine expression, lipid metabolism (based on **4a,b**) can be used to predict heterogeneity in progression dynamics for example associated with different outcomes. **(4b)** Finally, the knowledge inferred from serial sections using MSI/DSP may be routed back to label granuloma sub-types. Image classifiers can then be used to train on H&E or nuclear stain to learn and predict pathology and function from gross morphology. **(5a)** Variable features obtained from 1 to 3 are then re-probed on new tissue sections obtained from a larger cohort of subjects and through segmentation analysis or higher resolution targeted MSI. This targeted approach aims at the analytical validation of novel biomarkers discovered during the holistic profiling in 1.1–1.3. **(5b)** Intervention studies using agonists/blockers/metabolic agents or anti-leishmanial drugs to further establish causality associated with identified targets.

Granulomas are mononuclear cell-rich inflammatory foci formed around “indigestible” materials or pathogens and *Leishmania donovani* infection provides one of the best studied examples of experimental hepatic granuloma formation (Murray, 2001; Kaye and Beattie, 2016). Further, a transcriptional network enriched in genes for lipid metabolism were identified in uninfected bystander Kupffer cells that are responsible for parasite survival (Beattie et al., 2013). Interestingly, *L. brazililensis* was shown to affect host cell lipid metabolism (Alves-Ferreira et al., 2020). We show here as an example, a pipeline for the analysis of liver granuloma development following experimental visceral leishmaniasis and their associated lipid profile (using MSI) to help strategize therapeutic approaches. These methods are, however, generally applicable to immune microenvironments (Figure 1).

**FIGURE 2:**
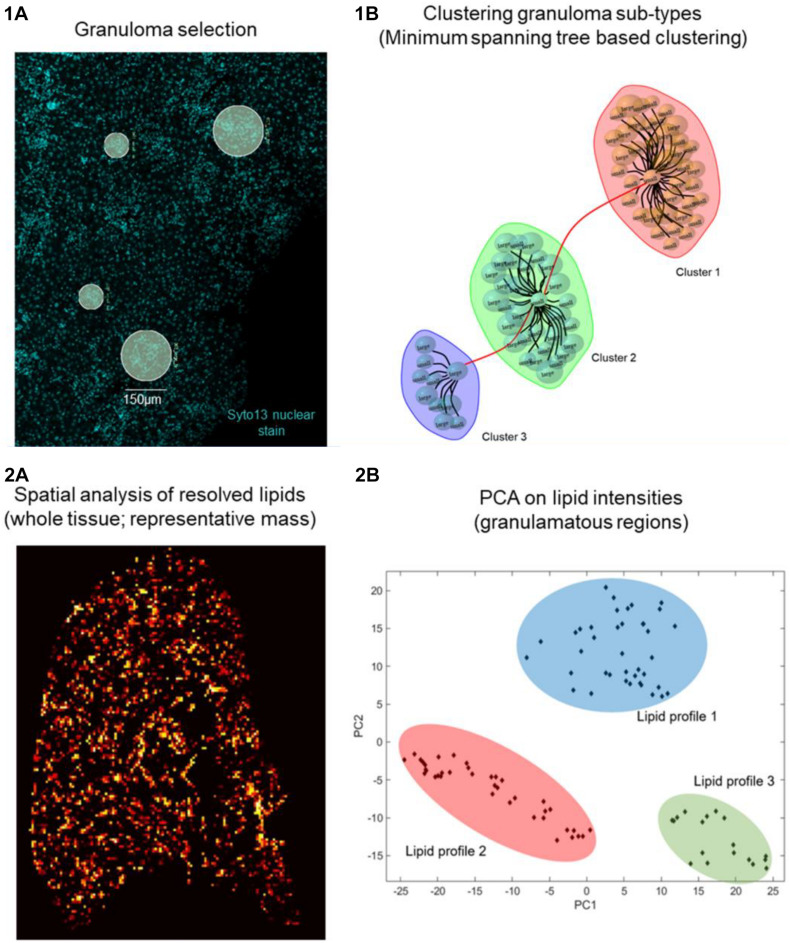
Preliminary data on *Leishmania* granuloma. Day 28 p.i. *L. donovani* infected C57BL/6 mice liver sections (*n* = 5) were **1A** stained with SYTO 13 nuclear dye and Nanostring Immune Cell Profiling Core and IO Drug Target Modules (protein panel, 26 oligo labeled antibodies). Eighty granulomas (75 or 150 μ diameter) were selected based on nuclear dye on a Digital Spatial Profiler (DSP, Nanostring). UV light cleaved ROIs from individual granulomas were collected in a 96 well plate, hybridized, and counted on an nCounter system **(1B)**. Distinct granuloma clusters as calculated based on edge betweenness of a minimum spanning tree representation of a granuloma weighted (Pearson’s correlation) graph object. **(2A)** Day 14 *L. donovani* infected mouse liver sections (*n* = 5) were analyzed by MSI. **(2B)** Principal component analysis (PCA) is used as an unsupervised approach to show variance in spatial distribution of all lipid masses from every pixel across all granuloma regions. The PCA image depicts highly intense pixels, which represent granulomas and contain different lipid profiles. A total of 20 granuloma ROIs per mouse liver section were analyzed. Subsequent PCA shows discriminative lipid profiles, which suggests that granulomas show heterogeneous lipid signatures.

Using GeoMx DSP, ROIs can be created on granulomas using established morphological stains. Next, to identify compositional differences between the granuloma microenvironment and the surrounding parenchymal tissue can be identified using MSI-guided spatial omics. As an exemplar, and in line with our proposed workflow depicted in Figure 1, we show preliminary results on aberrant spatially resolved protein and lipid profiles from granulomas in *L. donovani*-infected mice using DSP and MSI (Figure 2). High-level (low-plex) exploratory analysis of cellular composition of granulomas using targeted spatial protein profiling and MSI on serial sections can identify heterogeneity in ROI (Figure 2.1) composition and metabolic programming (Figures 1.1, 1.2 for strategy and Figure 2.2 as exemplary data). Such heterogeneity can then be used to explore more mechanistic questions using further iterations of targeted DSP and MSI, e.g., involving cell-specific segmentation (Merritt et al., 2020) in previously characterized ROIs and/or by casting a wider net to delineate heterogeneity at whole transcriptome resolution.

The structure of formatted omics data is often an *X*_i_*_j_* matrix where the *i* = *r*_1_, *r*_2_, *r*_3_, …, *r*_m_ representing the abstraction level of the measured object (e.g., individuals, single-cells, or ROIs) and *j* = *j*_1_, *j*_2_, *j*_3_, …, *j*_n_ representing the features of the measured object (which can be single nucleotide polymorphisms, mRNA counts, protein, lipid, or other macromolecular expression intensity). Data generated using DSP and MSI can be easily converted into a rectangular format to describe protein/RNA and metabolite levels as features (column) per ROI (row). Each omics field has developed tools and pipelines for curating and analyzing data, but they provide general ideas for analyzing this type of data (Robinson et al., 2009; de Wit et al., 2012; Butler et al., 2018). We briefly describe methods that can be used to analyze the spatial structure and distribution of multi-modal measurements.

### Clustering ROIs to Find Biological Communities

Features with low dispersion in the data are removed to exclude non-informative features (i.e., curse of dimensionality) which can reduce the distance between the data points. Variable features, as selected above, can be normalized to the number of nuclei or area of microenvironment. Features can then be scaled and used as candidates for dimensionality reduction by principal component analysis and this is generally recommended for both small and large datasets (MSI and single-cell RNA) in terms of accuracy and efficiency (Klerk et al., 2007; Sun et al., 2019). It is relevant to assume that heterogeneity in inflammatory microenvironments may be due to cellular activation or composition and that these discrete communities may be classified as discrete clusters. *k*-means clustering can be used to identify clusters that best model the data by selecting an appropriate “*k*” using the elbow method (Diday, 1994). Graph-based methods try to detect clusters by identifying nodes that are connected tightly using a distance metric. For example, one approach to this is to calculate the edge betweenness that assigns highest scores for edges that are traversed often to find shortest paths and, therefore, are assumed to connect discrete communities/clusters (Freeman, 1979; Brandes, 2001). Methodologies adopted in high dimensional scRNA sequencing datasets have shown that while there is hardly one clustering method that fits all problems (Lockett, 2020), when it comes to clustering scRNA data graph-based tools such as Seurat (Butler et al., 2018), SCANPY (Wolf et al., 2018), and PhenoGraph (Levine et al., 2015) are able to identify clusters very efficiently (Kiselev et al., 2019). Many algorithms (including non-graph based) have been used for scRNA-seq data [tabulated in Kiselev et al. (2019)] and may be borrowed for analysis of ROI-based high dimensional datasets. An important consideration here is that clustering works well for individual cell types but the signal obtained from a granuloma will be composite and may require additional de-convolution steps (Biancalani et al., 2020; Kleshchevnikov et al., 2020) to aid or inform clustering using publicly available or in-house scRNA sequencing datasets.

### Trajectory-Based Analysis

Granulomas may be similar or distinct, as well as asynchronous in their development (Albergante et al., 2013; Moore et al., 2013; Kaye and Beattie, 2016). This suggests that while the selected ROIs may be grouped as discrete clusters, they may also be related to each other in a continuous sense. In 2003, methods to order temporally sampled microarray data correctly by calculating minimum spanning tree of a complete graph were developed (Magwene et al., 2003). These methods have been extended and applied to high-dimensional single cell RNA datasets to infer a pseudotemporal ordering of individual cells. Single-cell transcriptomes show heterogeneity and imply asynchronicity in expression patterns. Thus, cells sequenced at a single time-point can be statistically ordered in pseudotime to reveal lineages (Trapnell et al., 2014). This is not restricted only to single lineages but can indicate the possible branching of trajectories. In the context of inflammatory microenvironments such as granulomas, trajectories can be applied to ascertain progression or maturation. Protein/RNA snapshots at multiple timepoints can further aid computational interpretation of microenvironment progression trajectory. The asynchronicity of granuloma formation and outcome may be mapped on to a pseudo-temporal space. Interpreting such information in the context of underlying spatial metabolism may indicate how the latter affects immune cell composition or activation score in a spatially resolved manner. Further, probabilistic methods of learning temporal trajectory have been described for single cell RNA data that can be applied for multi-modal ROI analysis (Campbell and Yau, 2016a) especially those that can incorporate prior knowledge in trajectory learning (Campbell and Yau, 2016b). At this stage, clustering information of lipid profiles (based on MSI) may be associated [co-analysis (Buescher and Driggers, 2016)] of exact serial sections) with the granuloma progression trajectory as inferred *via* protein expression data (DSP) to link metabolic profiles to granuloma progression (Figure 1.3).

Further, as both DSP and MSI retain exact spatial information, inferred ROI subtypes can be associated to metadata available from tissue morphology. Correlations found at this step to morphology and pseudotime ordered ROIs can then be applied to probe more mechanistic questions like how proximity to underlying morphology can impact immune cell activation or metabolism. This can be achieved by asking broader fundamental questions by informing mathematical models (Figure 1.4a) of granuloma development in parallel (Albergante et al., 2013; Moore et al., 2013; Siewe et al., 2017; Moyo et al., 2018) or by targeted experiments and interventions, e.g., an *ex vivo* biomarker validation study in a larger cohort (Figures 1.5a, 1.5b).

### Computational Image Processing

Statistically inferred ROI sub-types can be further explored at the single cell level by co-registering MS images with IHC of the entire tissue based on cell types or proteins identified earlier in the process. QuPath (Scupakova et al., 2020), an open-software tool for digital pathology image analysis was successfully aligned with single-cell MALDI-MSI data allowing for automated single-cell morphometrics with their detailed molecular profile (Scupakova et al., 2020). Using other specialized software like Strataquest (TissueGnostics StrataQuest, 2021) and Cell Profiler (Mcquin et al., 2018) it is then possible to identify mechanistic reasons for observed branching in pseudotime. In parallel, classification of ROIs in branched trajectories can be used to label ROIs for training convolutional neural networks to identify such clusters in routinely stained images (Shi et al., 2019). These can be used as a powerful research or diagnostic tool (Figure 1.4b).

## Conclusion

Spatially resolved immunometabolism advances our knowledge of different immune lineages and populations central to microbial infections but more specifically can help delineate pathogenesis in important infections such as tuberculosis (Silva Miranda et al., 2012), schistosomiasis (Hams et al., 2013), and leishmaniasis (Kaye and Beattie, 2016) where pathogen induced metabolic re-wiring of host cells has been implicated in the pathological progression of the disease (Wilson et al., 2019; Saunders and McConville, 2020). Recent dual RNA-seq studies evaluating host and pathogen transcriptional response simultaneously show that metabolic interplay between the intracellular pathogen and the host cell can determine microbicidal or permissive property of the microenvironment (Westermann et al., 2016, 2017; Russell et al., 2019). In addition, MSI based reconstruction of host–pathogen lipid metabolic responses has been shown for *Francisella* infection (Scott et al., 2017) and for specific glycolipids in tuberculosis lesions (Blanc et al., 2018). Further understanding of the heterogeneity in development, function, or progression of these granulomas in relationship to their tissue microenvironment requires further detailed *in situ* exploration of the relationship between host immune cells, pathogen niches, and local biomolecule (protein, lipid, and transcript) profiles.

## Data Availability Statement

The original contributions presented in the study are included in the article/supplementary material, further inquiries can be directed to the corresponding authors.

## Author Contributions

RT, SD, and ND designed and wrote the manuscript. GC and PO’T provided insights and feedback. PMK and RMAH reviewed the manuscript and are project leaders and corresponding authors. All authors contributed to the article and approved the submitted version.

## Conflict of Interest

The authors declare that the research was conducted in the absence of any commercial or financial relationships that could be construed as a potential conflict of interest.

## Publisher’s Note

All claims expressed in this article are solely those of the authors and do not necessarily represent those of their affiliated organizations, or those of the publisher, the editors and the reviewers. Any product that may be evaluated in this article, or claim that may be made by its manufacturer, is not guaranteed or endorsed by the publisher.
